# Automated Computer-Aided Detection and Classification of Intracranial Hemorrhage Using Ensemble Deep Learning Techniques

**DOI:** 10.3390/diagnostics13182987

**Published:** 2023-09-18

**Authors:** Snekhalatha Umapathy, Murugappan Murugappan, Deepa Bharathi, Mahima Thakur

**Affiliations:** 1 Department of Biomedical Engineering, SRM Institute of Science and Technology, Chennai 603203, India; 2 College of Engineering, Architecture, and Fine Arts, Batangas State University, Batangas 4200, Philippines; 3 Intelligent Signal Processing (ISP) Research Lab, Department of Electronics and Communication Engineering, Kuwait College of Science and Technology, Block 4, Doha 13133, Kuwait; 4 Department of Electronics and Communication Engineering, School of Engineering, Vels Institute of Sciences, Technology, and Advanced Studies, Chennai 600117, India; 5 Center of Excellence for Unmanned Aerial Systems (CoEUAS), Universiti Malaysia Perlis, Arau 02600, Perlis, Malaysia; 6 Department of Electronics and Communication Engineering, SRM Institute of Science and Technology, Ramapuram, Chennai 600089, India

**Keywords:** intracranial hemorrhage, deep learning models, classification, SE-ResNeXT, LSTM, Grad-CAM model, ResNeXT

## Abstract

Diagnosing Intracranial Hemorrhage (ICH) at an early stage is difficult since it affects the blood vessels in the brain, often resulting in death. We propose an ensemble of Convolutional Neural Networks (CNNs) combining Squeeze and Excitation–based Residual Networks with the next dimension (SE-ResNeXT) and Long Short-Term Memory (LSTM) Networks in order to address this issue. This research work primarily used data from the Radiological Society of North America (RSNA) brain CT hemorrhage challenge dataset and the CQ500 dataset. Preprocessing and data augmentation are performed using the windowing technique in the proposed work. The ICH is then classified using ensembled CNN techniques after being preprocessed, followed by feature extraction in an automatic manner. ICH is classified into the following five types: epidural, intraventricular, subarachnoid, intra-parenchymal, and subdural. A gradient-weighted Class Activation Mapping method (Grad-CAM) is used for identifying the region of interest in an ICH image. A number of performance measures are used to compare the experimental results with various state-of-the-art algorithms. By achieving 99.79% accuracy with an F-score of 0.97, the proposed model proved its efficacy in detecting ICH compared to other deep learning models. The proposed ensembled model can classify epidural, intraventricular, subarachnoid, intra-parenchymal, and subdural hemorrhages with an accuracy of 99.89%, 99.65%, 98%, 99.75%, and 99.88%. Simulation results indicate that the suggested approach can categorize a variety of intracranial bleeding types. By implementing the ensemble deep learning technique using the SE-ResNeXT and LSTM models, we achieved significant classification accuracy and AUC scores.

## 1. Introduction

An intracranial hemorrhage (ICH) can result from bleeding within the intracranial vault as well as bleeding in the brain parenchyma and in nearby meningeal spaces as a byproduct of bleeding within the intracranial vault. In the case of ICH, it is a fatal disease that requires prompt medical attention and comprehensive interventions at the right time. It is estimated that approximately ten percent of stroke deaths are caused by intracranial hemorrhages. There are approximately 40,000 to 67,000 cases of ICH per year in the United States, according to the American National Institutes of Health, based on a prevalence estimate of 24.6 cases per 100,000 person-years [1]. Additionally, hemorrhages in the brain account for 8 to 13 percent of all strokes that occur in the brain [2]. The number of patients at risk of death in this group is estimated to be 40%. The reasons for this may differ from patient to patient and can be based on one or more factors.

Firstly, it is very important to diagnose the type of acute ICH so that precise treatment can be provided. Ischemic cerebral hemorrhage can be classified into various types, such as Intraparenchymal Hemorrhage (IPH), Intraventricular Hemorrhage (IVH), Subarachnoid hemorrhage (SAH), Subdural Hemorrhage (SDH), and Epidural Hemorrhage (EDH). The majority of subdural hemorrhages are caused by bridging veins within the dura and the arachnoid membrane, and the bleeding is predominantly caused by the rupture of these veins [3]. Hemorrhage in the subarachnoid space, which often occurs as a result of bleeding from the cerebral artery, is commonly called a subarachnoid hemorrhage. An intraventricular hemorrhage occurs when bleeding occurs anywhere within a ventricle of the brain, and it is usually the result of another hemorrhage that has occurred previously. During a cerebral hemorrhage, blood may flow anywhere within the brain’s neural tissue, referred to as an intraparenchymal hemorrhage, while epidural hemorrhages can occur between the dura and the skull.

As a part of the process of managing the individual and making sure that they receive the best assessment, medication, and rehabilitation, it is necessary to determine the exact location of the bleeding and how it occurred in order to ensure the best possible care. A method is implemented in [4] that detects ICH in a faster way by using Convolutional Neural Networks (CNNs) and Long Short-Term Memory Networks (LSTMs). An experimental analysis of the RSNA 2019 dataset was conducted, and a weighted mean log loss of 0.04989 was obtained with 98% accuracy when comparing it with the RSNA 2014 dataset. The Grad-CAM technique is utilized in order to enhance the fast diagnosis of ICH by increasing the detection rate.

CNN is one of the most effective deep learning algorithms for diagnosing brain diseases due to its ability to learn and classify the features of complex images automatically [5]. Several researchers have used explainable Artificial Intelligence (AI) and deep learning techniques in their medical imaging applications [6,7,8,9]. A Computerized Tomography (CT) scan is the preferred method of diagnosis for brain disease because it provides a better spatial resolution compared to other forms of imaging. It is also more sensitive to detecting brain hemorrhages by visualizing the brain regions, which makes it more appropriate for diagnosing brain diseases [10]. It is a challenging issue for radiologists to determine the exact diagnosis of ICH from CT images, since the bleeding areas in the brain can also be easily misinterpreted as calcifications or stripping artifacts. Despite having the same ICH subtype, hemorrhages can be markedly different in size, shape, and location, even when the hemorrhage is exquisite. In this paper, we present a hybrid deep learning technique that is effective at detecting ICH specifically by its type for the purpose of assisting healthcare providers in the detection of ICH.

Intracerebral hemorrhage (ICH) can cause enduring cognitive impairment or even death, requiring immediate diagnosis and treatment. It is well known that ICH is a significant contributor to fatalities, resulting in an elevated mortality rate. A brain injury can cause death and paralysis if it is not detected early enough. It is possible to diagnose ICH using CT scans because they provide a reliable and non-intrusive imaging method. In patients presenting neurological symptoms that indicate ICH, skilled radiologists use CT images to identify any intracerebral hemorrhage. Reviewing a CT scan manually by a radiologist is both time-consuming and demanding. Patient deaths can result from urgent treatment requirements in combination with the severity of the condition. An automated system is thus needed to detect both ICH and its specific subtype, addressing the urgency of critical cases, increasing diagnostic speed, and assisting radiologists. In order for an automated tool to be effective in medical diagnosis, it must display high levels of accuracy.

SE-ResNeXT combines a Squeeze-excitation block with a Residual network and Inception network. This reduces the difficulty of training deep networks by incorporating shortcut connections between layers. In addition, Multi-Step LR (learning rate adjustments) and transfer learning are used to enhance the performance of the network. The model focuses on picking out the most significant features of the lesion and leaving out the less significant ones. The combined ResNet and Inception models form the ResNeXT model, which performs group convolutions. Finally, a hyperparameter called cardinality is used to manage each group in this ResNeXT model.

Grad-CAM interprets the model decision-making process. ICH lesions are visualized as heat maps, which enable a clear understanding of the lesions present. Through Grad-CAM, a trained DL model can be used effectively to classify ICH types. Finally, the Grad-CAM assists doctors and physicians in making decisions based on AI in massive datasets.

This present work aims to diagnose the ICH and its subtypes (IPH, IVH, SAH, SDH, and EDH) by implementing ensemble deep learning techniques. The key contributions of the proposed work are as follows:

- The CT scan images are preprocessed by resampling, downscaling, and cropping certain regions of the brain to gain fine-grained details about the type of ICH.
- A windowing technique is used on three layers (bone, brain, subdural) to create an image with better contrast.
- A fine-tuned ensemble convolutional neural network (SE-ResNeXT + LSTM) is proposed to classify the intracranial hemorrhage, and its performance is compared with various statistical metrics.
- Finally, Grad-CAM visualization is used to identify the region of interest in the CT scan images to identify the type of ICH.

A brief introduction to the proposed work and major contributions to the related field are presented in Section 1. A detailed review of the recent literature on the topic is presented in Section 2. Section 3 describes the methodology, including the database used, how the preprocessing steps are performed, and how the ResNet, SE-ResNeXT, and LSTM models are compared with the pre-trained models. The results of the proposed work are discussed in Section 4 after a comparison with the existing literature. Lastly, in Section 5, we report our conclusions about the present work.

## 2. Literature Review

The use of deep learning for medical image analysis has risen dramatically in recent years [11,12,13,14]. Automatic classification and detection of acute ICH using deep learning algorithms is presented in [15]. This work uses Artificial Intelligence (AI) to diagnose acute ICH, with CNN classifiers as the first stage, followed by Sequence Models 1 and 2 and recurrent neural networks with three-dimensional slices that are used to extract appropriate feature outputs for detecting acute ICH. In this study, researchers conducted a multi-class classification of ICH based on three datasets: Physionet-ICH, RSNA, and CQ500. According to the results obtained, EDH can be diagnosed with high sensitivity compared to other types of ICH. Additionally, the proposed work provides better classification accuracy using 2019 RSNA datasets than the other two datasets. Due to the complexity of the model and the lengthy training process, this work has significant limitations.

Barin et al. [16] proposed a hybrid CNN model by combining EfficientNet-B3 and Inception-ResNet-V2 for ICH diagnosis. However, the proposed work is limited to the overfitting problem, despite reaching 98% accuracy. A hybrid deep neural network was constructed in [17] to predict intracranial hemorrhage. CNNs and LSTMs are combined to implement systematic windowing. The features are extracted from the CT image (RSNA dataset) using a CNN with a 10-window slice input. LSTM models are trained to categorize ICH hemorrhage types based on the extracted features. Approximately 72,516 CT scan images are used for training, and 7515 and 7512 CT scan images are used for validation and testing. With an accuracy of 95.14%, sensitivity of 93.87%, and specificity of 96.45%, the proposed Conv-LSTM model outperforms the other models. However, selecting the optimal features for training the model is challenging.

A machine learning algorithm is presented in [18] to identify and classify ICHs. Tsallis entropy (TE) is used in conjunction with the grasshopper optimization algorithm (GOA) for the segmentation of CT images. A Dense Net 201 model is used to extract features, and an Extreme Learning Machine (ELM) is used to classify ICHs. The Physionet-ICH dataset is analyzed in this study to classify the ICH as IPH, IVH, SAH, SDH, or EDH. A DenseNet-ELM model showed the highest accuracy of 96.34% among all state-of-the-art ICH classification models. Due to the random initialization of weights and biases during training, this work involves high intricacy of hidden layers. In [19], EfficientNet’s deep learning technology is discussed in relation to the diagnosis of ICH. Furthermore, the Grad-CAM method and Hounsfield Unit (HU) are used to visualize the desired region. The RSNA dataset is used for experimental analysis, and results show 92.7% accuracy; however, the method it is computationally intensive.

A study conducted in [20] uses deep learning to classify ICH and its types on CT images. Real-time CT image analysis is conducted using a deep neural network (DNN) at the Burdenko Neurosurgery Center in Russia. To achieve precise classification, ResNext50 architecture is deployed with the Adam optimizer, which adaptively amends the channel-wise feature responses. This work classifies IVH with an accuracy of 89.3%. For real-time datasets, setting and generalizing hyperparameters like filter size and stride is challenging. In [21], ensembled deep neural networks for diagnosing ICH are introduced. In order to classify ICHs, the given image is preprocessed, and the data is augmented before applying an efficient B0 network. Lastly, class activation mapping is used to visualize the bleeding area. A major limitation of this study is the imbalanced distribution of ICH and its subtypes in the RSNA dataset. Despite data augmentation being performed to minimize the above-mentioned issue, high accuracy with low sensitivity is still achieved.

A CNN and Recurrent Neural Network (RNN) are combined to predict ICH and its subtypes [22]. A CT slice is preprocessed for two types of classification (ICH and non-ICH) as well as five different types (IPH, IVH, SAH, SDH, and EDH). In addition to identifying bleeding precisely, Grad-CAM is also used for this purpose. A slice- and subject-level automated algorithm is used to analyze all subtypes of ICH. Compared to 2D CT scans, it takes less time to process 3D CT scans. The AUC value for all the ICH subtypes is greater than 0.8. The study concentrates on data collection from Asian populations, which limits the algorithm’s generalizability. The training set has unequal amounts of data for abnormal and control groups. This affects the performance of the algorithm. In [23], ICH is classified using a deep neural network, built using the residual network 152 and local binary pattern-based features. The fusion-based feature extraction with deep learning (FFE-DL) model outperformed other models, with an accuracy of 96.6% in the classification of ICH. According to the results, the ResNeXT study achieved the same 96.6% accuracy as ours.

Tharek et al. [24] demonstrated that feed-forward CNNs could detect and classify ICHs and non-ICHs. In training and testing the network, 200 images from the public dataset were used to achieve an accuracy of 95%. Based on CT images, binary classification was performed to identify those with and without ICH. Their method combined hand-crafted features with deep learning models to extract useful feature information for accurate classification. They achieved 95.2% precision with their ResNeXT model. The feed-forward deep learning method achieved an optimal precision value of 96.43%. The Inception ResNet-v2, EfficientNet-B3, and hybrid models were used in [16] to classify different types of ICH. Using the RSNA dataset, the authors trained and tested their network models. As a result of the implementation of ResNet-v2, EfficientNet-B3, and hybrid models, they obtained 98.2%, 98.1%, and 98.5% accuracy, respectively. The highest accuracy was achieved by hybrid models (combined with Inception—ResNet-v2 and EfficientNet-B3).

A binary classification-based pre-trained MobileNet-v2 was used in [25] to detect brain hemorrhage. RSNA datasets were used to train the images, and Vietnam hospital datasets were used for testing. To highlight the areas suspected of being ICH, several preprocessing techniques were applied, including windowing and density-based spatial clustering of applications with noise (DBSCAN). Their results demonstrate a sensitivity and specificity of 99.2% and 80.2%, respectively. Since the authors used fewer datasets for testing, the model was less effective. It is necessary for the authors to obtain additional test data in order to improve their performance accuracy. Lee et al. [26] conducted a study using real-time CT images of ICH cases and healthy controls. Using deep learning algorithms based on the Kim–Monte Carlo algorithm, three types of ICH were classified: EDH, SAH, and IPH. An SAH accuracy rate of 91.7% was achieved in comparison to an overall accuracy rate of 69.6%. The major drawback of this study involves the need to validate the study by increasing the sample size.

The most commonly used diagnostic procedure for patients experiencing symptoms such as strokes or an increase in intracranial pressure is a CT scan of the head without contrast. According to the study’s findings, deep learning algorithms can be used to identify abnormalities in head CT images that require immediate treatment. Using metadata and CT scans from the RSNA project dataset, this paper develops, implements, and validates an ensemble convolutional neural network (SE-ResNeXT + LSTM and ResNeXT + LSTM) for diagnosing ICH. Two databases, RSNA and CQ500, are used in the proposed study to generate CT images of ICH. The RSNA dataset is used for training, and the CQ500 dataset is used for testing. Automated feature extraction and multi-class classification of ICH into different categories are performed using the ResNeXT-2D + LSTM model and SE-ResNeXT-2D + LSTM model. The severity of hemorrhages can be determined using Grad-CAM visualization. The performance of the proposed ResNeXT and SE-ResNeXT models in combination with LSTM is compared with pre-trained models such as ResNet 50, Inceptionnet-v3, Mobilenet-v2, VGG19, and DenseNet 121.

## 3. Materials and Methods

**Figure 1:**
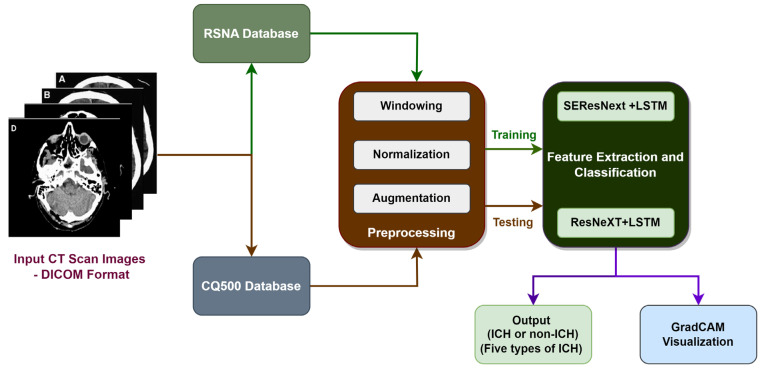
Block diagram of proposed work for the categorization of Intracranial Hemorrhage.

An overview of the proposed study for classifying various types of ICH is shown in Figure 1. In the proposed block diagram in Figure 1, the input CT scan images are obtained from the RSNA database, and the input CQ500 images are obtained from the Qure.ai database. As a training database, the RSNA database is used, while the Qure.ai database is used for testing purposes. A windowing technique is initially applied to the RSNA database in order to prepare it for further processing. There are three different kinds of windows that are utilized for preprocessing the images, namely a brain window, a subdural window, and a bone window. A windowing technique is applied in order to improve the RGB visualization of the data. Then, the dataset is normalized, and the process of data augmentation is carried out based on the normalized dataset. Using the image data generator API in Tensor flow V2.13.0, we were able to preprocess the testing dataset and augment it with additional data. Using two different deep learning algorithms, ResNeXT-2D + LSTM and SE-ResNeXT-2D + LSTM, two automated feature extraction techniques were used, and a multi-class categorization of different ICHs was performed. Additionally, the Grad-CAM algorithm was used in order to find the regions of interest in various types of ICH.

### 3.1. RSNA Database

This hybrid model is analyzed using two different kinds of datasets, namely RSNA [27] for the training process and CQ500 for testing and validation purposes. In this study, we used the RSNA2019 Brain CT Hemorrhage challenge dataset, collected from three institutions (Stanford University (Palo Alto, CA, USA), Universidade Federal de Sao Paulo (Sao Paulo, Brazil), and Thomas Jefferson University Hospital (Philadelphia, PA, USA). More than 60 neuroradiologists annotated the images in the dataset to group them into different categories. In total, there are more than a million images in the original dataset. The training set in the RSNA dataset contains 133,709 slices, while the test set contains 14,600 slices (Table 1). In the data, there are five different ICH types—EDH, IPH, IVH, SAH, and SDH—but the distribution of each is highly unbalanced. All the CT images are captured using the non-contrast-enhanced method and stored in DICOM format, with a pixel resolution of 512×512.

### 3.2. CQ500 Database

In this work, we used the CQ500 dataset for testing the proposed ICH classification system. The CQ500 database contains a maximum of 171,390 images of different types of brain images obtained from a total of 491 CT scans. These images include ICH (205 scans), fractures (40 scans), middle shifts (65 scans), mass effect (127 scans), and normal controls (54 scans), among other types of brain images. During the study, different slice thicknesses (0.625 mm, 3 mm, and 5 mm) of CT images were captured using GE and Philips CT imaging devices on subjects aged between 7 and 95 years old in India. The images were annotated into different categories by three senior neuroradiologists who are specialists in neurological imaging. This study’s dataset included data on every type of cerebral bleeding.

### 3.3. Hounsfield Scale

**Figure 2:**
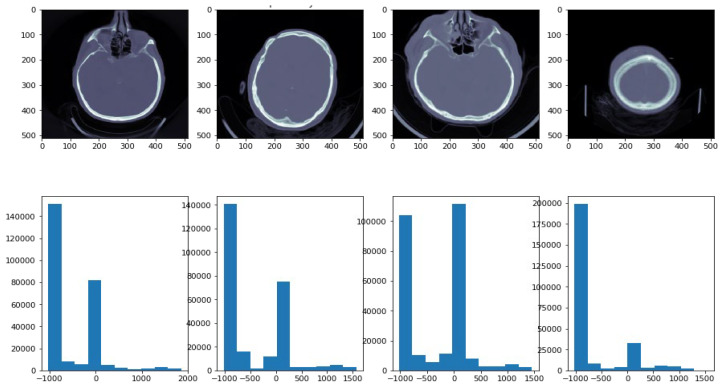
ICH images with their corresponding histograms. In the histogram, the x-axis demonstrates image intensity, while the y-axis demonstrates pixel count.

Grayscale in medical CT imaging is measured in Hounsfield Units (HUs). There are 4096 values (12 bits), and the scale ranges from −1024 HU to 3071 HU (zero is also a value). Using this scale, it is possible to correlate the attenuation of CTs with the density of tissues [28]. Using the “windowing” technique, CT images are enhanced based on their contrast. If two parameters are specified, the window level and the window width, it is possible to see the given window (mapped into the entire grayscale range (0 to 255)). A white color would be perceived if the value was greater than (l + w/2), whereas a black color would be perceived if the value was lower than (l − w/2). There are two important window settings for CT images: Bone window and Brain window. We used Python’s DICOM library, using command line APIs including Rescale Intercept and Rescale Slope. The rescaling of CT images to Hounsfield units was carried out in different steps using Python API, as shown in Figure 2. This method was used to determine the values of the histograms of the images. Figure 2 shows ICH images with their corresponding histograms.

### 3.4. Data Preprocessing Using Windowing and Augmentation

**Figure 3:**
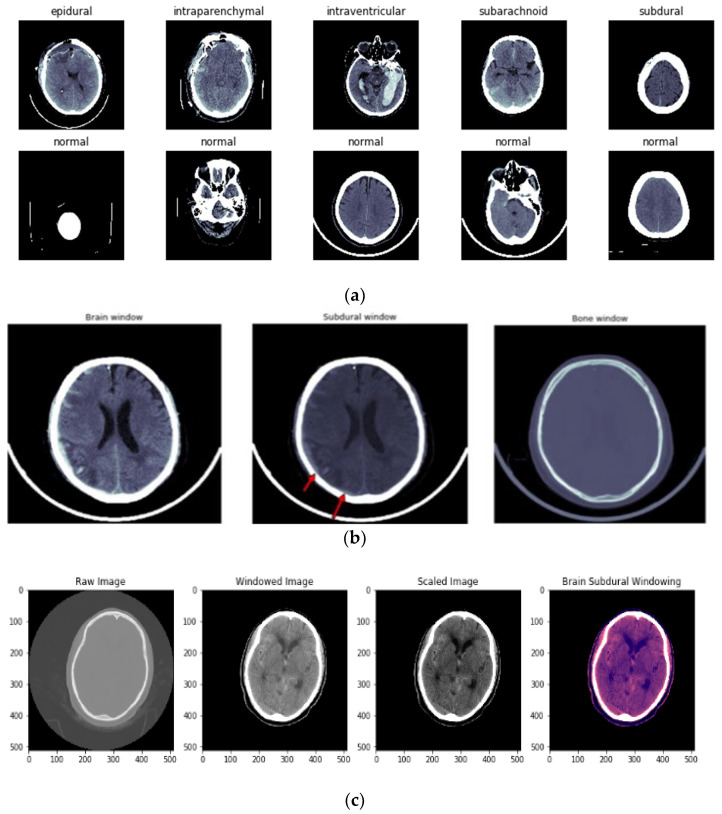
(**a**) Different types of ICH and normal images before windowing is applied. (**b**) Types of windowing used in preprocessing of ICH. First image shows the brain window, second image represents the subdural window, and third image indicates the bone window. (**c**) Image preprocessing using the windowing technique. First image shows the raw CT image, second image shows the windowing technique applied over the raw image, third image is a scaled image, and final image is brain subdural window applied image.

The first step in preparing the dataset was to convert intensities into HUs (Hounsfield Units). Next, three windows were implemented: brain, subdural, and bone windows (Figure 3b). Figure 3c illustrates the windowing techniques used for preprocessing ICH images.

In the preprocessing step, the contrast-adjusted image is obtained by applying the subdural window to the raw image. A training image with a size of (224,224,3) is provided to the network architecture to determine the optimum size for training. Eighty percent of the data used in the RSNA dataset is for training, and 20 percent is for testing. To validate the dataset, 10-fold cross-validation is performed.

**Figure 4:**
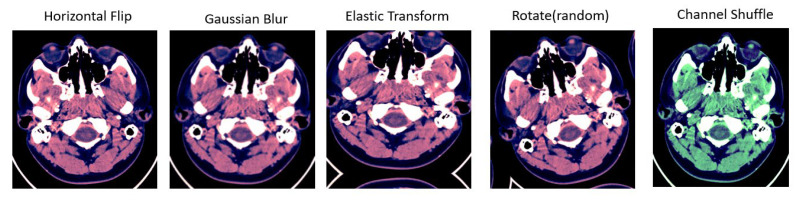
Different types of augmentation used for training data. First image shows the horizontal flip operation applied over the input image; second image is Gaussian blur operation used for augmentation; Elastic transform and rotation operation are applied in third and fourth images; fifth image shows the channel shuffling applied over the input image.

A technique of image augmentation was applied to the dataset in order to enhance the efficiency and robustness of the model training. We used Python’s (version 3.10) augmentation module and TensorFlow’s image data generator API (version V2.13.0) in this study. The selected augmentation techniques are described and illustrated in Table 2 and Figure 4.

### 3.5. Implementation Details

We use the Tensorflow–Keras framework on a server that has two NVIDIA Tesla V100 GPUs, with 32 GB of graphics memory each. As a first step, we need to scale the image to these dimensions: $\left(w\times h\right)$ = (224 × 224). Moments are estimated by the Adam optimizer, which has an exponential decay rate of (1, 2) = (0, 99) and a learning rate of 1.25 × 10^−5^. Using the Scikit-learn Python library, machine learning algorithms are used to pre-analyze the process. An Intel i7 processor-powered Windows 10 operating system with 64-bit OS and 16 GB RAM was used to execute Python codes.

#### 3.5.1. ResNeXT Architecture

The ResNeXT model shows the Next dimensions on top of ResNet. As a result of ResNeXT’s implementation, sub-modules such as Inception are used, the reaction time for each operation is calculated, and the products are concatenated at the end of each module. These sub-operations within the module are described in a new dimension called “cardinality”. By repeating sub-operations within a substantially lower encoding, ResNeXT simplifies module design. A major objective is to reduce the number of hyperparameters required by conventional ResNets. We combined the best characteristics of VGG Net and Inception designs. VGG Net is an architecture based on tiny kernels, which have repeated layers, and Inception is an architecture based on micro-networks. Each filter was employed in the same number of steps to obtain the same output size. The number of filters was doubled if the output’s spatial dimension was half. In this technique, the temporal complexity of each tier was maintained at the same level. Furthermore, expanding cardinality is critical for improving accuracy, and promoting cardinality is more effective than developing deep convolutional neural networks.

In ResNeXT, the hyperparameter “cardinality” indicates the number of paths found in each block. It is usually set to 32, with a width of 4 for the bottleneck d and a width of 128 for the group convolution. It has a computational complexity of 7.8 GFlops. The model uses the following tuning parameters:, weight decay (0.0001), momentum (0.9), and learning rate (0.1). The computational time required to execute the model is 164 ms or 1.7 s per mini batch.

#### 3.5.2. SE-ResNeXT Architecture

Squeeze-excitation (SE)-ResNeXT models are generated by applying the SE module to residual blocks. The result is the transformation of a single residual route into numerous residual pathways within the ResNet block. Both Visual Geometry Group Nets (VGG architectures) and ResNet demonstrate that stacking blocks of the same form can decrease hyperparameters while maintaining state-of-the-art performance [29]. Further, Google Net and Inception have demonstrated the benefits of fine network architecture using split transform mergers. As a result of combining these two excellent concepts, ResNeXT was developed [30]. ResNeXT, in contrast to GoogleNet, simply repeats the same substructure, resulting in the split transform merge being completed; at the same time, hyperparameters are not significantly increased. A residual block of ResNeXT can be used to create SE-ResNeXT by including the SE module. Due to its unique ability to reduce the parameters and weight of the network without sacrificing its performance, the Squeeze and Excitation Block was chosen for this present work. The SE block consists of three layers: a global pooling layer, two fully connected layers (FC), and two activation function layers (Sigmoid and ReLU). Due to the SE block’s regulation of the overall weight parameters, the relevant traits are strengthened while the insignificant ones are weakened. The hyperparameters used in SE-ResNeXT model are specified in Table 3.

#### 3.5.3. LSTM

In RNNs, LSTMs are specifically designed to deal with the issue of lack of long-term reliance. Even though this model is adapted to learn lengthy dependencies, it is difficult for the network to learn long-term information. Rather than a simple convolution operation, there are four layers, each of which interacts with the others differently. LSTMs are characterized by their cell state, which is a horizontal line that runs down the chain, with a few linear interactions between them. It allows data to pass through in its original form without being altered. Table 4 illustrates the architecture of ResNeXT and SE-ResNeXT that was used.

### 3.6. Performance Metrics of Ensemble Approach

A variety of metrics are used to prove the competence of the proposed work, including accuracy, sensitivity, specificity, F1 score, precision, and Binary Cross Entropy (BCE) loss. Based on the simulation results, it can be demonstrated that the suggested approach provides a reliable method for identifying and categorizing a variety of intracranial hemorrhages. In an ensemble deep learning approach, SE-ResNeXT and LSTM models produced high accuracy and F1 scores, indicating a strong and reliable performance. In this study, True Positives, True Negatives, False Positives, and False Negatives are abbreviated TP, TN, FP, and FN, respectively. In order to analyze the experimental data, the following metrics are examined.

#### 3.6.1. Accuracy

The accuracy (ACC) metric evaluates the classification outcome general accuracy and is determined by Equation (1).

(1) $$\mathrm{ACC}=\frac{\mathrm{TP}+\mathrm{TN}}{\mathrm{TP}+\mathrm{FP}+\mathrm{TN}+\mathrm{FN}}$$

The proposed methodology employs ACC as a statistical metric to assess the classification performance of ensemble deep learning algorithms for various types of ICH.

#### 3.6.2. Precision

The metric of precision (PREC) pertains to the ratio of accurately predicted positive classifications to all positive predictions and is mathematically derived using Equation (2).

(2) $$\mathrm{PREC}=\frac{\mathrm{TP}}{\mathrm{TP}+\mathrm{FP}}$$

It is proposed that precision be used as a statistical measure for evaluating the effectiveness of the ensemble deep learning models in categorizing the various subtypes of ICH by relying on the positive predictions made by the models as a means of evaluating their effectiveness.

#### 3.6.3. Sensitivity

The Sensitivity (SEN) or True Positive Rate (TPR) metric evaluates the ratio of accurate positive classifications to the total number of positive cases and can be calculated using Equation (3).

(3) $$\mathrm{SEN}=\frac{\mathrm{TP}}{\mathrm{TP}+\mathrm{FN}}$$

We use SEN as a statistical measure to assess the accuracy of our ensemble deep learning models in detecting various subtypes of ICH among the positive cases.

#### 3.6.4. Specificity

The metric of Specificity (SPEC) or True Negative Rate (TNR) relates to the ratio of accurately identified negative classifications to the total number of negative cases and is determined by Equation (4).

(4) $$\mathrm{SPEC}=\frac{\mathrm{TN}}{\mathrm{TN}+\mathrm{FP}}$$

In the proposed approach, SPEC is used as a statistical measure to evaluate the capacity of the ensemble deep learning models to precisely detect the absence of various subtypes of ICH among factual negative cases.

#### 3.6.5. F1 Score

The F1 score is a statistical measure that calculates the harmonic mean of PREC and recall (also known as SPEC). It is computed using Equation (5).

(5) $$F1-\mathrm{score}=\frac{2\cdot \mathrm{TP}}{2\cdot \mathrm{TP}+\mathrm{FP}+\mathrm{FN}}$$

The proposed methodology employs F1 as a statistical measure to assess the collective effectiveness of the ensemble deep learning algorithms in accurately classifying diverse subtypes of ICH, considering both precision and recall.

## 4. Experimental Results and Discussion

We used the CQ500 dataset and RSNA 2019 dataset to conduct our experiments. In CT scan images, the gradient-weighted Class Activation Mapping (Grad-CAM) visualization methodology proved effective in accurately detecting and localizing regions of interest, facilitating subtype identification. Additionally, the SE-ResNeXT and LSTM models were found to perform better than pre-trained models.

### 4.1. Grad-CAM Visualization Results

By using Grad-CAM’s backpropagation visualization method, we can gain insight into the training model’s classification decisions. Grad-CAM is used to identify specific regions within the input that influence the model’s decision-making mechanism. Grad-CAM uses the spatial knowledge acquired by LSTMs and SE-ResNeXTs to identify the distinctive regions critical to classification.

**Figure 5:**
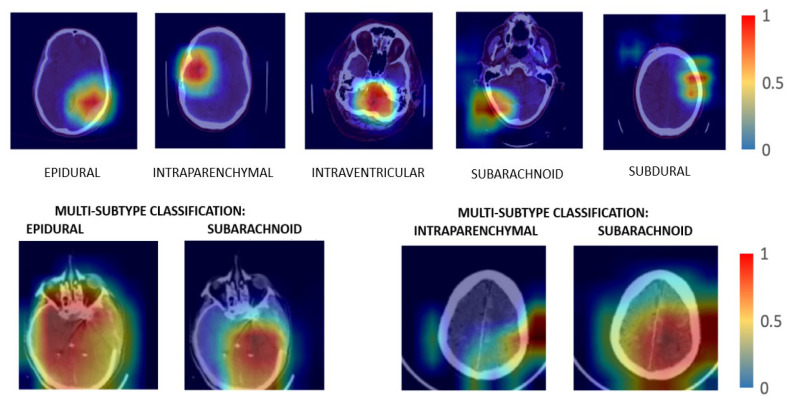
Grad-CAM visualization of subtypes of hemorrhage. The first image focuses on an epidural hemorrhage in the left parietal lobe. The second image concentrates on an intraparenchymal hemorrhage in the temporal right lobe. An intraventricular hemorrhage located in the posterior median lobe is shown in the third image. A subarachnoid hemorrhage present in right parietal lobe is shown in the fourth image. The fifth image represents a subdural hemorrhage in the left frontal lobe.

The Convolutional Neural Network (CNN) integrates feature extraction and classification into a unified system, employing a fully connected neural network in the classification module. The features that have been derived are converted into probability scores for every class. The segment with the highest score is then selected as the ultimate estimation or categorization result. Grad-CAM performs image classification and accurately identifies relevant regions using the gradients in the attribute map of the final convolutional layer. Figure 5 shows the visualization results for classifying ICH subtypes, namely Epidural, Intraparenchymal, Intraventricular, Subarachnoid, and Subdural. The first image focuses on an epidural hemorrhage in the left parietal lobe. The second image concentrates on an intraparenchymal hemorrhage in the temporal right lobe. An intraventricular hemorrhage located in the posterior median lobe is shown in the third image. A subarachnoid hemorrhage present in the right parietal lobe is shown in the fourth image. The fifth image represents a subdural hemorrhage in the left frontal lobe. Greater severity of the hemorrhage was present in the epidural, subarachnoid, and intraparenchymal regions of the brain.

The proposed methodology integrates ensemble deep learning models with Grad-CAM visualization to identify distinct areas of interest in CT scan images associated with each ICH subtype. The visualization results can facilitate a more precise categorization of each subtype based on its specific characteristics and locations. As a result, the proposed methodology enhances understanding and identifies and categorizes diverse forms of ICH with greater accuracy and effectiveness.

### 4.2. Binary-Cross-Entropy (BCE) Loss

We selected the Binary-Cross-Entropy (BCE) loss metric for this study. The selection is appropriate since the issue involves a multi-label classification of cerebral hemorrhages, where a single image may encompass multiple categories. The output layer implements sigmoid activation functions coupled with BCE loss in order to address this situation. This combination facilitates the model’s ability to assign probabilities to the different subtypes of hemorrhage in an autonomous manner, allowing for precise classification of various hemorrhage types within a single image. Equation (6) computes BCE loss.

(6) $$H_{p}(q)=-\frac{1}{N}\sum_{i=1}^{N}y_{i}\cdot \log\left(p\left(y_{i}\right)\right)+\left(1-y_{i}\right)\cdot \log\left(1-p\left(y_{i}\right)\right)$$

where the input sample is denoted as $y_{i}$, the probability of the input sample is denoted as $p\left(y_{i}\right)$, and the total number of samples is denoted as *N*.

**Figure 6:**
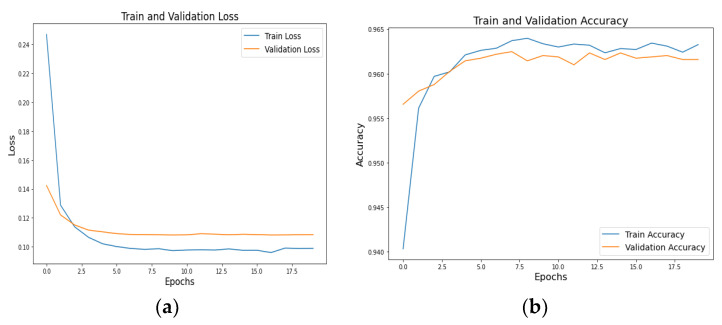
Training and validation (**a**) loss and (**b**) accuracy graphs of ResNeXT-2D + LSTM.

Graphs depicting the loss and accuracy of ResNeXT-2D + LSTM at various epochs are shown in Figure 6, with (a) representing the loss graph and (b) representing the accuracy graph. As the number of epochs increases, the loss diminishes, whereas the accuracy increases. A graphical representation incorporates both training and validation results, demonstrating the model’s potency throughout the process. Training and validation loss mean values of 0.10 and 0.12 demonstrate the model’s ability to reduce errors. A training accuracy of 0.961 and a validation accuracy of 0.960 show the model’s efficiency in classifying the numerous subtypes of ICH accurately. According to the findings, the proposed concept is successful, as evidenced by the model’s high accuracy and low loss ratios. Thus, the model is able to diagnose and classify different subtypes of ICH, which suggests its effectiveness.

**Figure 7:**
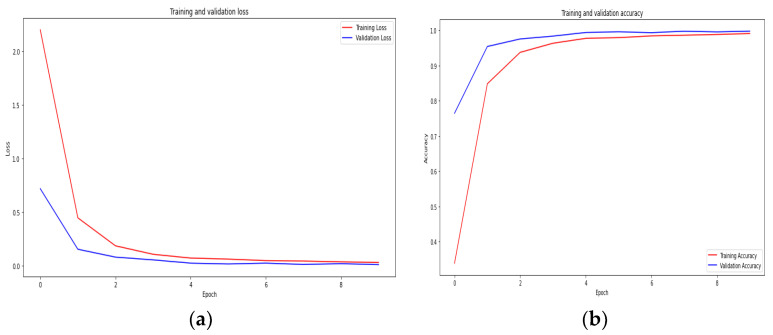
Training and validation (**a**) loss and (**b**) accuracy graphs of SE-ResNeXT-2D + LSTM.

A graphical representation of Figure 7a loss and Figure 7b accuracy of (SE) ResNeXT-2D + LSTM at various epochs is shown in Figure 7. Based on the graphical representations, there is a negative correlation between the number of epochs and the loss, and a positive correlation between the number of epochs and the accuracy. A steady decline in loss and an increase in accuracy is seen as the number of epochs increases from 0 to 9. In the graph, the training and validation outcomes illustrate the model’s effectiveness. Mean values of 0.01 and 0.03 are obtained for the training and validation losses, respectively. In terms of accuracy scores, the model categorizes ICH subtypes with exceptional precision, scoring 0.979 for training and 0.980 for validation. In addition to providing further evidence of the feasibility of the concept, this study also provides evidence of its efficacy. Based on the SE ResNeXT-2D model, various subtypes of ICH can be identified and classified with exceptional accuracy and minimal loss.

### 4.3. Confusion Matrix Analysis

**Figure 8:**
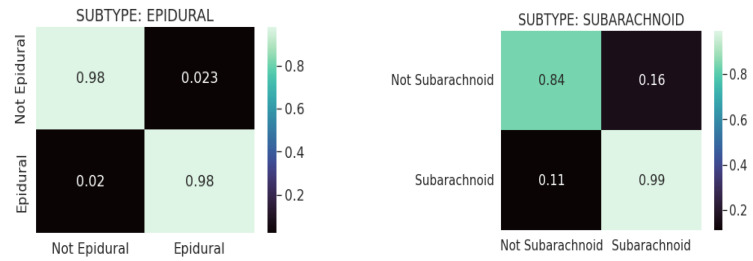

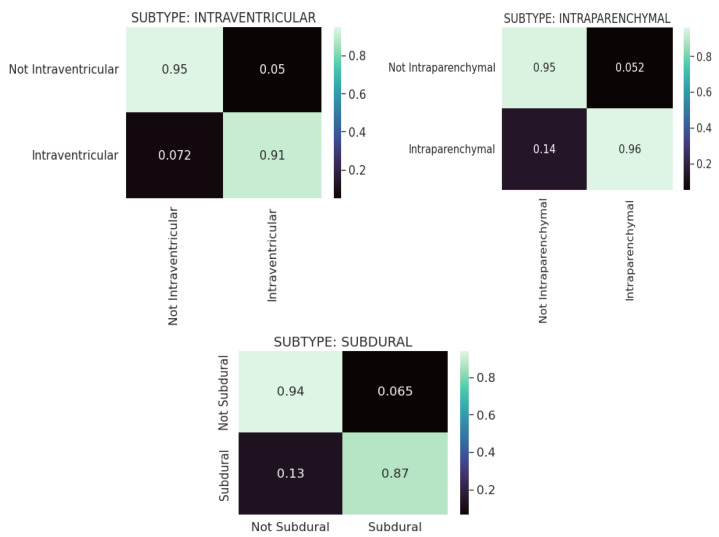
Confusion matrices for CQ500 (test) dataset using REsNext-2D + LSTM for each of the 5 subtypes of hemorrhage.

Figure 8 shows the confusion matrices for the CQ500 (test) dataset using ResNeXT-2D + LSTM for each of the five subtypes of hemorrhage. Across both the non-epidural and epidural subtypes, the model achieved a classification accuracy of 0.98. Regarding the subarachnoid subtype, the model achieved 0.84 accuracy for non-subarachnoid classifications and 0.99 accuracy for subarachnoid classifications. In the context of intraventricular subtypes, the model achieved a classification accuracy of 0.95 for non-intraventricular subtypes and 0.91 for intraventricular subtypes. Among non-intraparenchymal cases, the model achieved a classification accuracy of 0.95, while intraparenchymal cases attained a classification accuracy of 0.96. The model classified subdural cases with a classification accuracy of 0.94 and non-subdural cases with a classification accuracy of 0.87. Overall, the model showed excellent capability of categorizing intracranial hemorrhages accurately.

**Figure 9:**
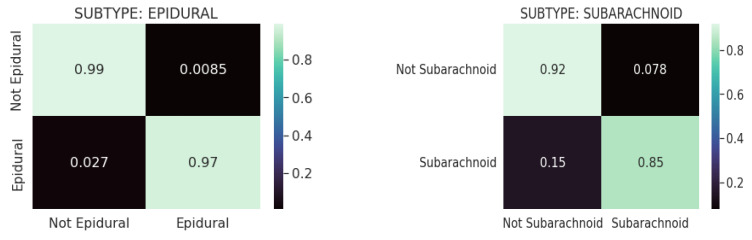

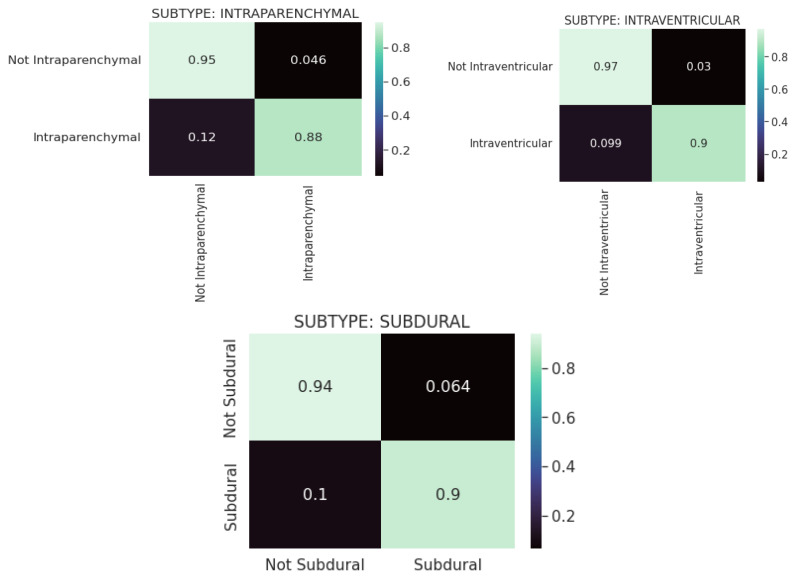
Confusion matrices for the CQ500 (test) dataset using SE-ResNeXT-2D + LSTM for each of the 5 subtypes of hemorrhage.

The confusion matrixes in Figure 9 are displayed for each of the five hemorrhage subtypes in the CQ500 (test) dataset using (SE) ResNext-2D + LSTM. Based on the model, the epidural subtype has a classification accuracy of 0.99 for non-epidurals and 0.97 for epidurals. Under the subarachnoid subtype, the model achieved a classification accuracy of 0.92 for the non-subarachnoid subtype and 0.85 for the subarachnoid subtype. This model determined a classification accuracy of 0.95 for non-intraventricular subtypes and 0.88 for intraventricular subtypes for intraventricular classifications. In intraparenchymal cases, the model achieved a classification accuracy of 0.97, whereas in non-intraparenchymal cases, the model achieved a classification accuracy of 0.09. In this study, the model was evaluated for its ability to detect subdural tumors. Non-subdural cases were classified with a classification accuracy of 0.94, while subdural cases were classified with a classification accuracy of 0.90. Based on the results, the ResNeXT-2D architecture with SE is capable of accurately classifying intracranial hemorrhages.

### 4.4. AUC-ROC Score

**Figure 10:**
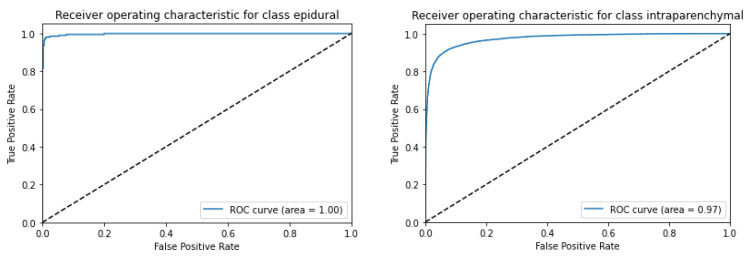

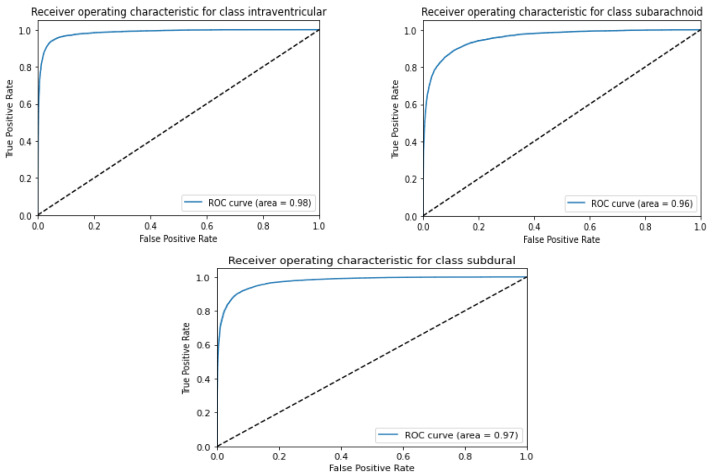
AUC-ROC score plot for CQ500 (test) dataset using ResNexXT-2D + LSTM for each of the 5 subtypes of hemorrhage.

Figure 10 depicts the AUC-ROC score plots for the CQ500 (test) dataset using ResNeXT-2D + LSTM for each of the five subtypes of hemorrhage. ROC curves were generated for epidural, intraparenchymal, intraventricular, subarachnoid, and subdural categories. Based on the receiver operating characteristic (ROC) curve, epidurals have an area under the curve of 1; intraparenchymal, intraventricular, subarachnoid, and subdural have an area under the curve of 0.97; and subdurals have an area under the curve of 0.97. This implies that ResNeXT-2D + LSTM can distinguish with precision between the various subtypes of intracranial hemorrhage, indicating exceptional efficacy in epidurals.

**Figure 11:**
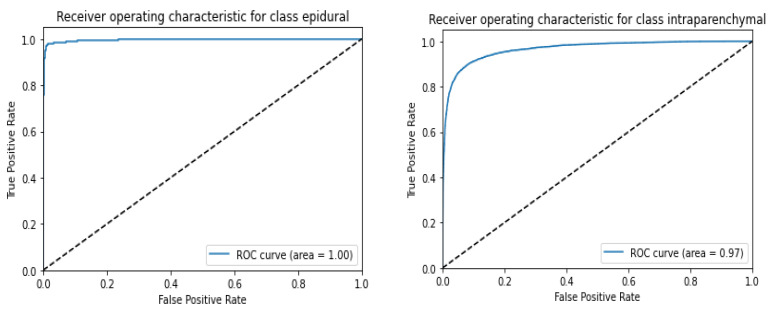

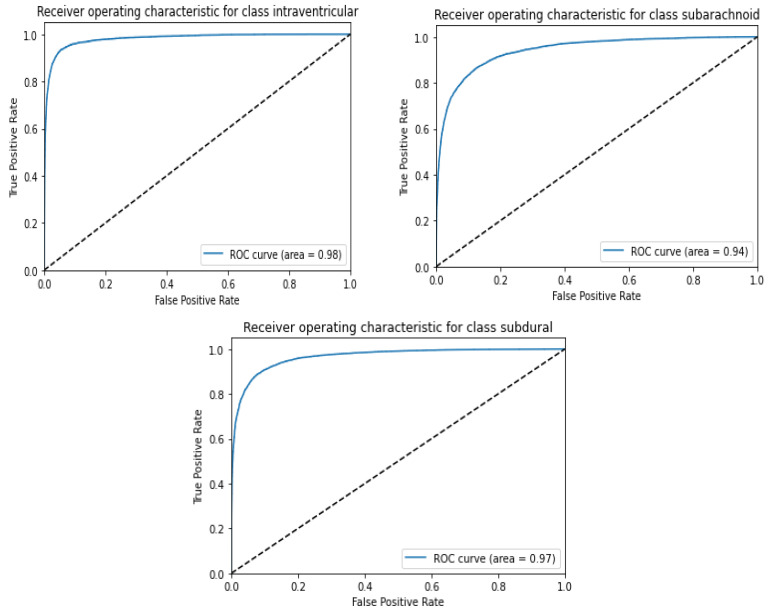
AUC-ROC score plot for CQ500 (test) dataset using (SE) ResNeXT-2D + LSTM for each of the 5 subtypes of hemorrhage.

Figure 11 shows the AUC-ROC score plot for the CQ500 (test) dataset utilizing ResNeXT-2D for each of the five subtypes of hemorrhage. A series of ROC curves was generated for epidural, intraparenchymal, intraventricular, subarachnoid, and subdural categories. Epidurals had an area under the ROC curve of 1, intraparenchymals had an area under the ROC curve of 0.97, intraventriculars had an area under the ROC curve of 0.98, subarachnoids had an area under the ROC curve of 0.96, and subdurals had an area under the ROC curve of 0.97. These findings highlight the remarkable discriminatory capacity of the (SE) ResNeXT-2D + LSTM architecture to detect distinct categories of intracranial bleeding. The model’s high AUC-ROC scores indicate its ability to distinguish between various classes. A notable achievement is the flawless classification of the epidural subtype. Based on the results, the proposed approach is capable of accurately categorizing various forms of hemorrhage.

It was demonstrated in Table 5 that the SE-ResNeXT + LSTM architecture was able to achieve excellent results across a number of statistical metrics (performance measures). The results of this study demonstrated that the epidural subtype had good discriminative capacity, which was evidenced by a value of 1 for the AUC. There were noteworthy levels of precision, recall, F1 score, and accuracy in all subtypes tested, proving that the method has the ability to accurately categorize a wide variety of hemorrhage types. There were a number of factors that indicate the reliability and resilience of this model, including its AUC of 0.99, its precision of 0.96, its recall of 0.98, its F1 score of 0.97, and its accuracy of 94%. This study confirms that the proposed approach is effective in accurately identifying and categorizing intracranial bleeding through the use of SE-ResNeXT + LSTM algorithms to identify and categorize bleeding.

**Figure 12:**
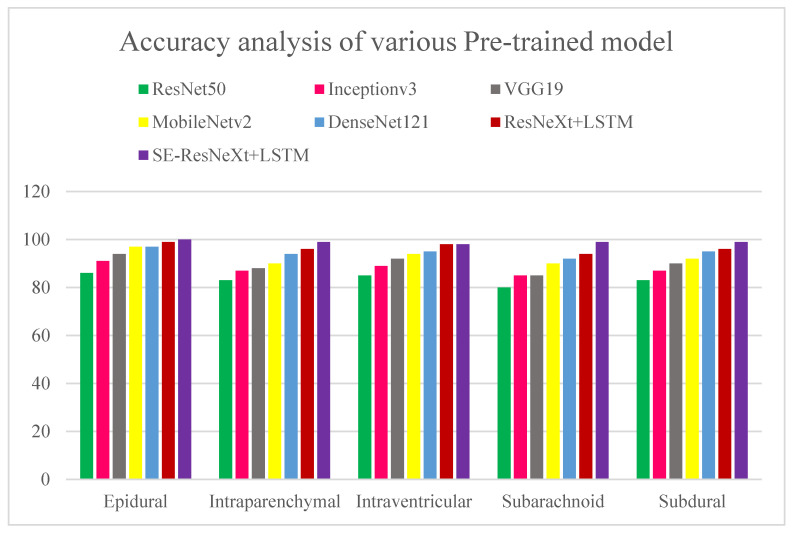
Accuracy analysis of various pre-trained models for diagnosing ICH and its types. The x axis represents the types of ICHs, and the y-axis represents the percentage of accuracy.

**Figure 13:**
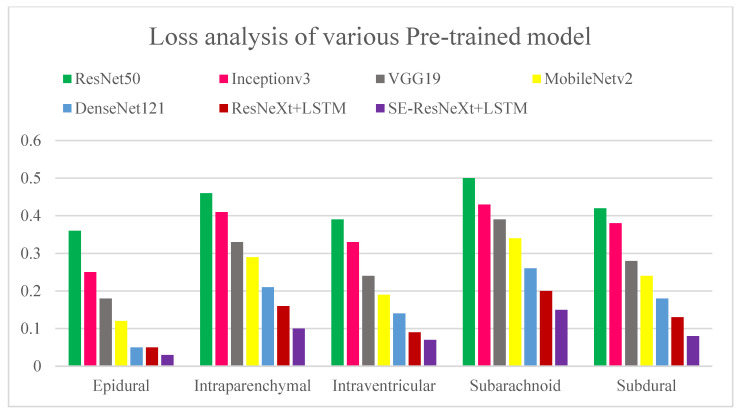
Loss analysis of various pre-trained models for diagnosing ICH and its types. The x-axis represents the types of ICHs, and the y-axis represents the detection loss.

Various pre-trained models to test for ICH are presented in Figure 12 and Figure 13 along with their accuracy and loss metrics. Several models are evaluated in the figures under consideration, including ResNet50 [31], Inceptionv3 [32], VGG19 [33], MobileNetv2 [34], and DenseNet121. Figure 12 illustrates the superior accuracy value achieved by the proposed ensembled approach in relation to other models used for diagnosing various kinds of ICHs. It was found that the ResNet50 model has the lowest accuracy score of 80% when it comes to predicting subarachnoid hemorrhage. According to ResNet50 and Inceptionv3, both models exhibit a loss score of 0.43 for predicting subarachnoid and subdural ICHs. The SE-ResNeXT + LSTM model achieved an average accuracy of 99% when it comes to diagnosing intracranial hemorrhages, regardless of the type of intracranial hemorrhage that was present. By observing the low loss of epidural ICH with the proposed model compared to the previous models, the competence of the proposed model is proven.

A cost-sensitive and query-by-committee active learning approach for intracranial hemorrhagic screening was proposed in [35]. As compared to previous studies, it was evaluated on a significantly larger pixel-wise labeled dataset, and its results were used to develop the model by marking real-world data. This study shows that cost-sensitive active learning can be used to improve major medical databases. Several quantifiable benchmarks were used to assess the accuracy of the community model of four patch-based FCNs (Fully Connected Networks), and the model received an accuracy rating of 90%. Using deep learning algorithms, nine critical anomalies were independently identified on CT scans of the head in [36]. Natural language processing (NLP) was used to identify intraparenchymal, intraventricular, subdural, extradural, subarachnoid, and subarachnoid hemorrhages in the study participants, as well as calvarial fractures. According to the algorithm, an image can be assigned a confidence score between 0 and 1, and each confidence score confirms the presence of each of the nine findings below in descending order of probability. Patients suffering from symptoms that might indicate a stroke or an increase in intracranial pressure most often undergo non-contrast CT scans of the head. A deep learning algorithm may be helpful for detecting abnormalities in head CT images that need prompt medical attention, according to the study’s findings. In most studies, binary classification and trinary classification of ICH are used. However, in our proposed study, the ResNeXT model had good accuracy for subtypes such as epidural (97%), intraventricular (94%), subarachnoid (85%), and subdural (91%). In epidural, intraparenchymal, intraventricular, subarachnoid, and subdural cases, SE-ResNeXT displayed better accuracy than ResNeXT, at 99%, 98%, 98%, 97%, and 99%, respectively. SE-ResNeXT achieved an average accuracy of 94% compared to ResNeXT (91.8%) and DenseNet 121 (92%).

Data acquired from different geographic regions distinct from the training dataset were used as external validation to assess the generalizability of the model. The proposed study utilized RSNA datasets obtained from three geographical locations for training, namely the UK, Brazil, and Philadelphia, and CQ500 datasets were obtained from Indian populations for testing. Thus, the proposed ensemble model classified epidural, intraventricular, subarachnoid, intra-parenchymal, and subdural hemorrhages with an accuracy of 99.89%, 99.65%, 98%, 99.75%, and 99.88%, respectively.

Several researchers have applied the SE-ResNeXT and ResNeXT models in various pathologies, such as ophthalmic diseases and gliomas. Fundus images are used in the automated diagnosis of multiple ophthalmic diseases based on the SE-ResNeXT model. Ho et al. evaluated the model in the Retinal Fundus Multi-Disease Image Dataset (RFMiD) and achieved an ROC area of 0.9586 [37]. Based on an SE-ResNeXT network, Li et al. classified 12 different retinal pathologies based on color fundus images and obtained an AUROC of 0.95 [38]. According to Linqi et al., a framework was developed to classify gliomas using the SE-ResNeXT network and ResNeXT model. Using the BraTS2017 dataset, they achieved 97.45 and 91.35% accuracy for SE-ResNeXT and ResNeXT, respectively [39]. Furthermore, SE-ResNeXT and ResNeXT models have been used in automated COVID-19 detection using chest radiographs, obtaining 99.32% accuracy in binary classification using the SE-ResNeXT model [40].

In this study, there were several limitations: The study encountered difficulties with managing large amounts of data. Due to the nature of the challenge, our computational methodology was slow to complete the task. The accuracy and AUC ratings of all models were below expectations. Therefore, we intended to show the discrepancy in empirical performance between older and newer networks. Due to the long training period, experimenting with other loss functions (such as weighted cross-entropy) as well as up-sampling and resampling strategies were difficult. Additionally, the models were only run for a limited number of epochs before achieving satisfactory results. In addition, it was difficult to conduct any other analyses (due to training time constraints) to ascertain whether certain sections of the pipeline should be deleted or inserted to better understand how it behaves. In the RSNA database, CT scans in DICOM format are stored in amounts up to 180 GB. Simpler models can be utilized for training purposes by using smaller chunks of data.

The computational complexity of the work can be summarized as follows: an SE-Res NeXt-2D + LSTM model and a ResNeXt-2D + LSTM model were run in Google Colab for 10 and 12 h, respectively. As compared to the ResNeXt-2D + LSTM model, the proposed SE-ResNeXt-2D + LSTM model takes less computational time and has fewer test losses. In Colab in a cloud environment, we ran pre-trained models on Intel Core I5 processors with 32 GB RAM. Optimizing the weights, tuning the model, and quantizing the data could reduce the computational complexity of the network.

It will be necessary in the future to employ a method that can analyze large amounts of data quickly and efficiently. There is a possibility of developing 3D model architectures to read 3D DICOM data as quickly as possible. Enhanced classification techniques will satisfy the needs of bigger clinical trials and implementations. To facilitate easy and fast medical use, end-to-end deep learning methodologies can be employed. An ensemble deep learning model and Grad-CAM visualization are combined to pinpoint specific regions of significance within CT scan images associated with each subtype of Intracerebral Hemorrhage (ICH). As a result of the visualization, a more accurate classification can be achieved based on the unique characteristics and positions of each subtype. Thus, the proposed approach contributes to an enhanced understanding of ICH and improves the efficiency and precision of identifying and classifying various types of ICH.

## 5. Conclusions

An ensembled deep learning technique (SE-ResNeXT + LSTM) is proposed for the detection and evaluation of intracranial hemorrhages. Moreover, this study compares the performance of CNN networks in two different databases, RSNA and CQ500. A comparison is made between the performance of these deep learning models and the performance of pre-trained models, such as ResNet 50, Inception V3, VGG19, Mobile Net V2, and Dense Net 121. According to the simulation results, the suggested approach is capable of categorizing different types of intracranial bleeding. An ensemble deep learning approach using LSTM and SE-ResNeXT models achieved significant accuracy and AUC scores, demonstrating dependable classification. Grad-CAM visualization is effective in identifying areas of significance in computed tomography scan images, facilitating the recognition of distinct hemorrhage categories. Additionally, the SE-ResNeXT + LSTM model demonstrated superior performance in accuracy, loss, and AUC metrics in comparison with various pre-trained models, confirming its appropriateness. In the future, large amounts of data need to be analyzed in a fast and efficient manner. Implementing 3D model architectures enables the rapid reading of 3D DI-COM data. A new generation of classification techniques will cater to the needs of more complex clinical trials and implementations. As a result of the proposed methods, precise and dependable diagnoses can be made, which can aid physicians and improve patient outcomes. Deep learning–based models have been widely accepted in the medical community and could be beneficial for decision-making, patient treatment, and overall health.

## Figures and Tables

**Table 1:** Training and testing datasets of RSNA dataset.

ICH Type	Training Set	Testing Set
EDH	2761	384
IPH	32,564	3554
IVH	23,766	2439
AH	32,122	3553
SDH	42,496	4670
Total	133,709	14,600

**Table 2:** Augmentation description table.

Operation	Description
Horizontal Flip	Flip the input image horizontally
Gaussian Blur	Blur the input image using a Gaussian filter with a random kernel size
Elastic Transform	Elastic deformation of images
Rotate	Rotate the input image by a random degree
Channel Shuffle	Randomly rearrange channels of the input RGB image

**Table 3:** Hyperparameters used in SE-ResNeXT model.

Learning Rate, LR	0.6
Epoch	200
Layer	50
Drop out	0.2
Crop Pct	0.875
Momentum	0.9
Interpolation	Bicubic

**Table 4:** Architecture of ResNeXT and SE-ResNeXT.

Layers	Output Shape	Kernel Size and Details
Convolution 2D	112×112	7×7 conv, stride 2
Max Pooling 2D	56×56	3×3 max−pool,stride 2
Conv Block (2)	56×56	$\left[1\times 1.128\;3\times 3.128,C=32\;1\times 1.256\right]\times 3$
Conv Block (3)	28×28	$\left[1\times 1.256\;\;3\times 3.256,C=32\;1\times 1.512\right]\times 4$
Conv Block (4)	14×14	$\left[1\times 1.512\;\;3\times 3.512,C=32\;1\times 1.1024\right]\times 6$
Conv Block (5)	7×7	$\left[1\times 1.1024\;3\times 3.1024,C=32\;1\times 1.2048\right]\times 3$
SE-Block (OPTIONAL)	7×7	Squeeze and Excitation Block×1
Classification	1×1	7×7 (global average−pool)
Layer	5	Fully Connected Dense Layer,Softmax

**Table 5:** ResNeXT + LSTM model results for various performance measures.

Subtype Name	AUC	Precision	Recall	F1 Score	Accuracy
Epidural	0.99	0.72	0.97	0.83	97%
Intraparenchymal	0.99	0.97	0.97	0.97	98%
Intraventricular	0.98	0.92	0.96	0.94	98%
Subarachnoid	0.99	0.95	0.99	0.97	97%
Subdural	0.99	0.99	0.98	0.98	99%
**Average**	**0.99**	**0.96**	**0.98**	**0.97**	**94%**

## Data Availability

Not applicable.
